# Finnish and Swedish riding school pupils' motivation towards participation in non-riding education

**DOI:** 10.3389/fspor.2023.1232428

**Published:** 2023-10-20

**Authors:** Lina Nyberg, Tanja Linnavalli, Elke Hartmann, Mirjam Kalland

**Affiliations:** ^1^ Faculty of Educational Sciences, University of Helsinki, Helsinki, Finland; ^2^ Diversity, Multilingualism and Social Justice in Education, Faculty of Educational Sciences, University of Helsinki, Helsinki, Finland; ^3^ Department of Animal Environment and Health, Swedish University of Agricultural Sciences, Uppsala, Sweden

**Keywords:** riding school, equestrian education, horsemanship, motivation, self-determination theory, basic psychological needs

## Abstract

Many horse enthusiasts have insufficient knowledge about horse behavior and welfare (BW) and learning and human-horse communication (LC), which poses a risk for both horse welfare and human safety. The main objective of this study was to investigate why riding school pupils participate or do not participate in non-riding education in BW and LC, using Self-determination theory (SDT). SDT posits that the quality of motivation is related to the individual's basic psychological needs. A convenience sample of 568 riding school pupils from Finland and Sweden completed an online questionnaire. The results showed that forty percent of the riding schools offered education in BW, and thirty-two in LC. Twenty-seven percent of the respondents participated in education in BW, and twenty-five in LC at their riding school. The respondents were autonomously motivated to participate in education, i.e., they would participate because it is interesting and personally important. Perceived needs satisfaction at the riding school predicted autonomous motivation to participate. Education was offered to a greater extent in Swedish riding schools and Swedish respondents participated more often, as well as experienced more autonomous motivation, relatedness and competence satisfaction compared with Finnish respondents. To our knowledge, this study is the first to explore riding school pupils' motivation towards non-riding education.

## 1. Introduction

For over a decade, researchers have raised concerns about equestrians' and horse enthusiasts’ insufficient evidence-based knowledge of various aspects of horse behavior and welfare (BW) and learning theory and human-horse communication (LC) (1–3). For example, many horse owners and caretakers have difficulties in recognizing signs of clinical health problems and behaviors indicative of negative affect or distress (4–7). The prevalence of equipment-related lesions in horses, recorded during equestrian competitions, indicate that many riders either are unaware of, or are neglecting horse welfare (8, 9). Further, studies indicate that horse enthusiasts, horse owners, and even professionals have significant gaps in knowledge of terminology, understanding of, as well as application of learning theory, which is a widely acknowledged theory of how animals learn (10–13).

The consequences of the lack of such knowledge and failure to meet the needs of the horse may be far-reaching and severe, both for the horse and the human. Horse-related human accidents top the accident and injury statistics in many countries (14) and inappropriate horse management practices have a considerable and wide-ranging effect on horses' physical and psychological wellbeing [for a review, see Hemsworth et al. (15)]. Besides significant welfare risks for both dyad members, the human-horse communication may be impeded, leading to poor performance and less enjoyability of the activity for both horse and human (16–18). One of the main reasons suggested for these shortcomings is insufficient or inadequate education (both theoretical and practical) that is not grounded in latest scientific evidence (12, 13, 19, 20). Indeed, research indicates that education in riding is based on tradition, not science (21–24), and puts little focus on interpretation and consideration of horse's behavioral and emotional responses to human signaling (25).

Riding schools are one of the most important educational platforms, as most riders in Finland and Sweden receive their basic education there. For example, of the one thousand riding stables in Finland, approximately five hundred function as riding schools (26). Riding schools in Finland and Sweden are very similar due to their common background and structure (23, 27–29), however, schools in Finland are almost exclusively run by private companies, whereas most of the Swedish schools are run by non-profit riding clubs (30). In both countries, many riding schools are affiliated to the Equestrian Federation in their respective country, establishing a link between riding schools and organized sports. The federations conduct quality assessment of the affiliated riding schools and provide education for youth leaders, who often run pony clubs at the schools (31, 32).

Riding schools provide lessons in riding to beginners as well as advanced riders of all age groups (23, 30, 31, 33), whereas non-riding lessons in horsemanship are provided less (30, 31, 34). In Finland and Sweden, horsemanship is commonly conceptualized as knowledge and skills about the horse in a broad sense, although excluding riding (35, 36). Non-riding education in horsemanship is often arranged outside riding lessons in separate courses led by youth trained by the national federation (28, 33). These courses are mainly aimed at children and teenagers. In Finland, the courses focus on basic knowledge of horses' fundamental needs (eg., forage, social contact with conspecifics), skills in preparing the horse for riding (grooming, tacking up and leading), and first aid (37). In Swedish riding schools, non-riding education in horsemanship has traditionally involved basic knowledge about and caretaking of the horse (28). Today, most riding schools in Sweden include at least one non-riding lesson per semester for all riding groups (38), although the contents of these lessons remain unclear.

The present study was part of an ongoing Swedish research project that aims to map current education and pedagogical tools to improve riding schools' knowledge base and to facilitate the integration of scientific advances into riding school practice. The main purpose of the current study was to explore riders' motivation, namely why they would, or would not, participate in non-riding education in BW and LC offered at riding schools. Motivation is at the core of human behavior, as it is the process that activates behavior, both in terms of power and direction (39). Thus, an individual may be inclined to do something to a higher or lesser degree. Further, the origin or quality of motivation differs (40). Often, the quality of motivation is explained as dichotomous, either stemming from an innate interest and enjoyment of the behavior (intrinsic motivation) or from the outside, when something else than the behavior itself is the reason (extrinsic motivation). Conversely, an individual may lack motivation altogether. Whichever the origin of motivation, several factors influence its activation and continuity: on the one hand, personal factors such as beliefs, and on the other hand, social and environmental factors (41).

One of the most popular motivation theories is Self-determination theory (SDT), which is a metatheory composing six mini-theories (42). Two of these, organismic integration theory (OIT) and basic psychological needs theory (BPNT) were utilized for the present study, as they focus on the complexity of motivation and how it is influenced by the environment.

According to BPNT, humans have three basic psychological needs; autonomy, competence, and relatedness (42–44). *Autonomy* refers to a sense of volition, willingness, and acting because one has freely chosen to do so. *Competence* concerns a sense of accomplishment, effectiveness, and capability. The third need, *relatedness*, refers to a sense of significance, belonging, and connection with others. An environment that supports the needs by, for example, allowing for choice, providing appropriate challenges, and encouraging social cohesion, predicts wellbeing (45) and facilitates *autonomous motivation* (46). Autonomous motivation is specified within OIT as volitional engagement in a behavior, i.e., when an individual perceives the behavior as fun, enjoyable, and interesting (*intrinsic* motivation), as a part of one's own identity (*integrated* regulation) or as personally important and valuable (*identified* regulation) (47). Thus, one may willingly and happily choose to do something that is not so fun, interesting, or enjoyable if the reason for doing so has been internalized.

**Figure 1:**
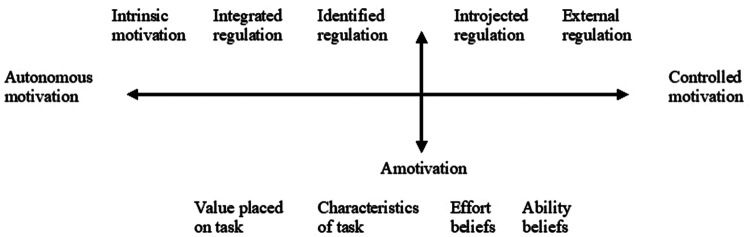
The continuum of motivation in line with Self-determination theory.

OIT focuses specifically on the internalization process, i.e., the process of taking in extrinsic reasons as internally regulated, that is, stemming from oneself (42, 43, 48). According to the theory, people tend to internalize practices and values in their social environment, specifically when their psychological needs are supported. If the internalization process is successful, autonomous motivation develops in the forms of integrated or identified regulation. If, however, the process of internalization is only partially successful, one acts due to inner pressure, such as guilt (*introjected* regulation), and if the process fails, one acts due to external pressure, for example to avoid punishment (*external* regulation), or do not act at all (*amotivation*). Introjected and external forms of motivation are conceptualized as *controlled* motivation. Hence, motivation is situated on a continuum of ascending autonomy, where least autonomous is external regulation and most autonomous is intrinsic motivation (Figure 1).

There is a vast body of evidence supporting the notions of BPNT and OIT and their interconnectedness. Meta-analyses have confirmed motivation as situated on a continuum (49, 50), although the distinct nature of integrated regulation was not confirmed in the latter, as it correlated too closely with identified regulation. Furthermore, meta-analyses have established that the more autonomous the motivational regulation, the more positive the outcomes. For example, intrinsic and identified regulation were associated with greater wellbeing and performance among students, as compared to introjected and specifically external regulation (46). In this meta-analysis, introjected regulation was weakly predictive of, and external regulation was unrelated to wellbeing and performance. A slightly different pattern was found among exercising adults, as identified and integrated regulation predicted the most positive outcomes, followed by intrinsic motivation (51). Introjected and external regulation were generally not associated with exercise behavior.

Satisfaction of the basic needs for autonomy, competence, and relatedness have repeatedly been connected to the different regulations of motivation (52). Thus, external regulation had no association with perceived needs satisfaction while introjected, identified, and intrinsic regulations were associated in an ascending manner. Integrated regulation was not included in this meta-analysis. Amotivation, on the other hand, has been linked to frustration of the basic needs as well as dysfunctional outcomes in both the physical education context (53), and the educational setting in general (52).

Amotivation is usually treated as a single component, however, it has been argued to be of a multidimensional nature (54, 55). The authors note that treating amotivation as a multifaceted construct could be an important addition to OIT, as it deepens the understanding of the reasons and related antecedents of the lack of motivation. Hence, students may experience amotivation in academic contexts due to four distinct reasons (54). The student may see no value or personal interest in the task (*value placed on the task*) or experience the task as boring and disengaging (*characteristics of the task*). Further, the student may feel unwilling to invest in the effort needed (*effort beliefs*), or experience that one is not able to do it (*ability beliefs*).

All four subdimensions of amotivation, i.e., ability, effort, value of task, and characteristics of task, were connected to non-adaptive outcomes and lack of social support (54). Further, within the physical education domain, effort beliefs and value placed on task were connected to the intention not to participate in further physical education (56). In a slightly different version of the multidimensional scale of amotivation, capacity (ability) beliefs were reported as the most important reason for adults' choice not to exercise (57).

Research on riders' motivation indicate that initial involvement with horses relates to intrinsic reasons, such as an innate liking of horses (58–60), whereas continued engagement with horses for leisure or sports activates both intrinsic and identified regulation of motivation. Mitchell (61), who used Self-determination theory, found that American leisure riders were intrinsically motivated to ride, that is, they ride for the fun and enjoyability of the activity. In terms of identified regulation, social aspects of the activity seemed to be most common (61). Being involved in the riding school community was the main reason for Swedish young riders’ participation at riding schools (62), and social reasons, alongside the possibility to ride, was the main reason for American college and university students' involvement in equestrian sport clubs (63). Sharing the experience and having fun with friends were the most prominent reasons also for mixed-aged Australian leisure and athlete riders, regarding participation in equestrian sport (58). The social aspects extend also to the horse. Elliot (59) and Bornemann (60), who investigated riders and horse owners in the UK, both report that the possibility to interact and establish a relationship with the horse were main motives for participation in equestrian activities. Besides social aspects, motives of the identified type include learning and relaxing (64). Wu et al. (64) investigated horse enthusiasts in the UK, riding school pupils included, and reported that wanting to learn was more common for riders under 30 years of age, whereas the desire to relax was more common for older riders. Although these studies give important indications about horse enthusiasts' motivation for involvement in riding activities, none investigated participation in non-riding education.

The basic psychological needs have not received much attention from researchers interested in the equestrian domain. Bornemann (60) explored English horse owners' motivation towards owning a horse, applying among others the basic psychological needs theory (BPNT), one of the six mini-theories within SDT (42). The results suggest that ownership was initiated by the need for autonomy and to a slightly lesser degree relatedness, as riding school lessons or loaning of horses did not sufficiently satisfy the needs. The respondents wanted to make their own decisions regarding the horse, how to care for it and “use” it, and to establish a bond with the horse. Caring for the horse was a crucial part of ownership, satisfying both the need for competence and relatedness. Interestingly, the needs for relatedness and competence were in this data related to the horse, not other humans. In fact, other humans were mostly perceived as frustrating of the needs, for example by giving negative feedback or restricting respondents' practice. The respondents preferred environments in which advice was not given, and in which they had the freedom to learn through experience and trial and error.

In conclusion, education has been proposed as a means to address riders’ insufficient knowledge about, and skills to handle the horse. Specifically, knowledge in horse welfare, behavior, learning, and human-horse communication have been identified as important to ensure horse welfare and rider safety. Engagement in such education requires motivation, which, in line with Self-determination theory, is linked to perceived psychological needs satisfaction. While research indicates that riders are autonomously motivated to ride, i.e., they ride because it is fun and personally important, no evidence exists regarding motivation towards non-riding education.

### 1.1. Aim and research questions

The main purpose of the study was to explore why riders would, or would not, participate in non-riding education offered at riding schools. To our knowledge, this cross-national study is the first to explore both riders’ motivation towards non-riding education, and perceived needs satisfaction at the riding school, and as such, contributes to the theory by exploring a new field. Further, the results are relevant to researchers and practitioners within the equestrian domain. Understanding the reasons behind riding school pupils' motivation and lack of motivation may influence decisions regarding provision of education. The research questions were:

1. To what extent do Finnish and Swedish riding schools offer, and to what extent do riding school pupils participate in education in two non-riding subjects, i.e., horse behavior and welfare (BW) and learning and human-horse communication (LC)?
2. What are the motivational and amotivational regulations of Finnish and Swedish riding school pupils regarding participation in education in BW and LC?
3. What is the relationship between perceived psychological needs satisfaction at the riding school and autonomous motivation to participate in education in BW and LC?
4. Are there differences between Finnish and Swedish respondents?

## 2. Materials and methods

### 2.1. Questionnaire

This cross-sectional study was based on an online questionnaire that collected quantitative data from 409 Finnish and 159 Swedish riding school pupils (568 in total). The questionnaire consisted of four sections: (1) respondents' demographics, (2) participation in education in horse behavior and welfare (BW) and learning and human-horse communication (LC), (3) motivation towards participation in BW and LC, and (4) perceived basic psychological needs satisfaction at the riding school.

*In section 1*, the respondents were asked to answer 7 closed questions on demographics, e.g., age group, how long they have been riding/taking care of horses, and whether they have competed during the last two years. Competition was added because it stands as a measure for involvement and progression in the hobby.

*In section 2*, the respondents were asked to report back on whether they participated in non-riding education in BW and LC at the riding school and outside the riding school during the last two semesters (autumn 2021 and spring 2022). They also reported whether education in BW and LC are offered at the riding school. This section included 4 close-ended multiple-choice questions and one optional open-ended commentary field.

*In section 3*, the respondents were asked to report back on reasons for participating, and reasons for not participating in education in BW and LC at the riding school where they ride. It was emphasized that the respondents could answer the questions also in case the riding school did not offer such education. The respondents reported on a 5-point Likert scale (1 = strongly disagree, 3 = partly agree, 5 = strongly agree). This section included in total 29 close-ended statements and one optional open-ended commentary field.

The respondents' motivational regulation was measured using a 15-item scale, developed from the Sport Motivation Scale II (SMS-II) (65) and the Behavioral Regulation in Sport Questionnaire (BRSQ) (66). The SMS II and BRSQ are based on Self-determination theory and were designed to assess respondents' quality of motivation towards sport. The SMS-II has 3, and the BRSQ has 4 items measuring each construct of motivational regulation (intrinsic motivation, integrated regulation, identified regulation, introjected regulation, external regulation, and amotivation). For this study, 3 items measuring the constructs of intrinsic, integrated, identified, introjected, and external regulation were chosen. The target group and the nature of the activity (participating in non-riding education) influenced the choice of included items. An example of the statements is: “I participate because it reflects who I am”. The items measuring amotivation were not considered suitable for this study, and thereby excluded.

The respondents' amotivational regulation was measured using a 14-item scale, developed from the Academic Amotivation Inventory (AAI) (54). The AAI has 4 items measuring each construct; value placed on task, task characteristics, effort beliefs, and ability beliefs, and has been used to assess students' multidimensional nature of amotivation. For this study, 3 items per construct that best suited the target group and activity were chosen, and one was excluded. This decision was made to keep the questionnaire as short as possible. An example of the statements is: “I do NOT participate because, for me, it holds no interest”. Research suggests that finances may be a barrier for equestrians' involvement in the activity (58, 59). Thus, one item was included to measure inability to pay for non-riding education, and one item to measure unwillingness to pay for education in BW and LC.

*In* *section 4*, respondents were asked to report back on how they perceive the riding school's general milieu during the last semester (spring 2022). The respondents answered on a 5-point Likert scale (1 = strongly disagree, 3 = partly agree, 5 = strongly agree). This section included 24 close-ended statements, two of which measured riding school pupils' experience of horse welfare at the riding school and were treated as unrelated to perceived needs satisfaction, and one optional open-ended commentary field.

The respondents' perceived satisfaction of the needs for autonomy, competence, and relatedness were measured using the 21-item scale Basic Psychological Need Satisfaction at Work Scale BPNS-W (67–69). The BPNS-W includes statements for satisfaction and frustration of autonomy (7 items), competence (6 items), and relatedness (8 items). One item was added to measure competence (“I have great difficulties handling some horses at the riding school”), resulting in 22 items for the scale. The decision to add an item measuring competence was two-fold. First, the original BPNS-W has an unequal number of items per construct, which has been priorly criticized (70), therefore the aim was to balance the number of items. Second, the horse has previously been identified as important for riders' perceived needs satisfaction (60), therefore an item with reference to the horse was considered relevant.

The scales used to measure motivational regulation, amotivational regulation, and basic needs were translated from English to Finnish and Swedish by the first author. The translations were checked by the author's supervisor and another person fluent in all three languages. During the translation process, the items were adjusted to fit the context of education at the riding school. The questionnaire was piloted among a group of nine Finnish and three Swedish riding school pupils to ensure comprehension of questions and overall structure and layout.

### 2.2. Procedure

Riding school pupils in Finland and Sweden, aged 15 years and older, were invited to participate in the online questionnaire, available from May 2022 to September 2022 at the online platform of the University of Helsinki (https://elomake.helsinki.fi). The Finnish questionnaire was distributed in May 2022 and September 2022 by email to all riding schools in Finland that are affiliated to the Equestrian Federation of Finland (206 in total). The Finnish questionnaire was also made accessible on the equestrian federation's website and social media, and in several informal Facebook groups related to riding. The Swedish questionnaire was distributed via the Swedish research project's website, horse-related websites, and in several informal Facebook groups related to riding. Further, it was distributed by email to ten riding schools of different size and location.

Data collection was anonymous, based on informed consent, and no personal data was collected such as name, email, or address. The respondents were informed that they could discontinue responding to the questionnaire at any time without consequences. The questionnaire study was in full compliance with Finnish and Swedish regulations on ethics in research involving humans and its accomplishment was not subject to notification according to the Finnish National Board on Research Integrity TENK (71) or the Swedish Ethical Review Act (72).

### 2.3. Data analysis

The data was analyzed using the statistical program IBM SPSS Statistics 28 for Windows (IBM Corporation, New York, USA). The central tendency and normality of the data were inspected. In case the criteria for normality were not met, non-parametric tests were used. No missing data was found.

#### 2.3.1. Creation of variables

As the scale used for measuring respondents' motivational and amotivational regulation in this study was developed from three different scales, an exploratory factor analysis (EFA) was conducted to determine the covariance among the motivational and amotivational variables. The principal axis factoring (PAF) analysis with direct oblimin rotation (73) and Kaiser normalization suggested six factors, including three factors for motivational regulation: intrinsic/identified, integrated, and external/introjected, and three other factors for amotivational regulation: task/value, effort, and ability. The variable external/introjected was named “controlled motivation” in agreement with Self-determination theory (42). One item representing introjected regulation (“I participate because I feel better about myself when I do”), and one item representing money issues (“I cannot afford to participate”), did not load with any other items, therefore they were excluded. See Supplementary material S1 for the factor loadings and communalities for the motivation and amotivation variables.

The variables for perceived autonomy, competence, and relatedness satisfaction were created according to the scale provided by Deci et al. (67), Ilardi et al. (68), and Kasser et al. (69). The added item measuring competence (“I have great difficulties handling some horses at the riding school”) correlated significantly with the other competence items (coefficients ranging between.15 and.41, *p* = <.001), therefore, it was included in the sum variable measuring competence.

A variable measuring perceived horse welfare at the riding school was created using the two additional questions in section 4 of the questionnaire. To perform the statistical tests, new variables for total needs satisfaction (an average of autonomy, competence, and relatedness), autonomous motivation (average of intrinsic/identified and integrated regulation variables), total amotivation (average of task/value, effort, and ability) were created. The descriptive statistics including Cronbach's alpha for all sum variables are shown in Table 2.

#### 2.3.2. Statistical tests

Internal consistency (Cronbach's alpha) was computed for all variables. All variables demonstrated acceptable to good reliability, except controlled motivation (*α*.64). Comparisons between groups were conducted with the Chi-square test of independence and Mann-Whitney tests. Spearman's correlation was used to examine the relationships between groups and between variables. A multiple linear regression analysis was used to estimate the relationship between autonomous motivation and total need satisfaction while controlling for demographic variables. No violations to the assumptions for normality of residuals, homoscedasticity, and multicollinearity were found. The significance level was set at *p* < .05.

## 3. Results

### 3.1. Respondents

The respondents included five hundred and sixty-eight (*N* = 568), almost exclusively female (96,5%) riding school pupils. The demographics are summarized in Table 1.

**Table 1:** Summary of the demographics of the Finnish and Swedish respondents.

	Finnish		Swedish		Full sample	
	Count	%	Count	%	Count	%
Gender						
Female	392	95.8%	156	98.1%	548	96.5%
Male	8	2.0%	3	1.9%	11	1.9%
Other	3	0.7%	0	0.0%	3	.5%
Prefer not to say	6	1.5%	0	0.0%	6	1.1%
Age group						
15–25	90	22.0%	38	23.9%	128	22.5%
26–35	82	20.0%	17	10.7%	99	17.4%
36–45	98	24.0%	29	18.2%	127	22.4%
46–55	87	21.3%	44	27.7%	131	23.1%
56 or older	52	12.7%	31	19.5%	83	14.6%
Hobby years						
5 years or less	80	19.6%	13	8.2%	93	16.4%
6–10 years or not regularly	97	23.7%	34	21.4%	131	23.1%
11–20 years	107	26.2%	38	23.9%	145	25.5%
21 years or more	125	30.6%	74	46.5%	199	35.0%
Hobby frequency						
Once a week or less	170	41.6%	76	47.8%	246	43.3%
Twice a week or more	239	58.4%	83	52.2%	322	56.7%
Competing						
Yes	156	38.1%	54	34.0%	210	37.0%
No	253	61.9%	105	66.0%	358	63.0%
School size						
<10 horses	63	15.4%	21	13.2%	84	14.8%
11–20 horses	230	56.2%	47	29.6%	277	48.8%
21 > horses	114	27.9%	89	56.0%	203	35.7%
I don’t know	2	0.5%	2	1.3%	4	.7%

### 3.2. Participation in education in horse behavior and welfare (BW) and learning and human-horse communication (LC)

**Figure 2:**
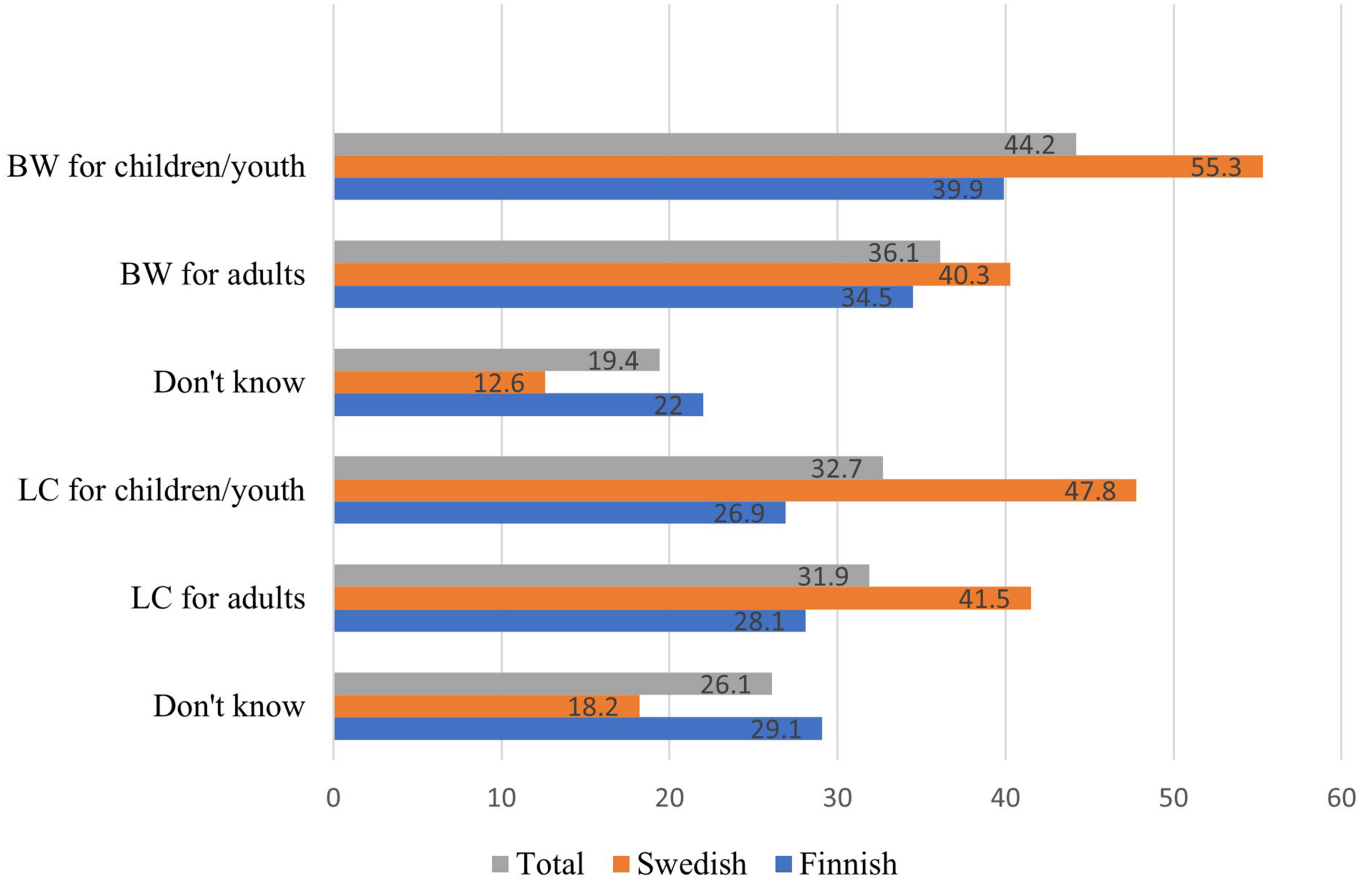
Illustration of education in BW and LC offered at the riding school. Numbers are presented in percent (%).

Of all respondents (*N* = 568), 40% reported that education in BW, and 32% reported that education in LC is offered at the riding school where they ride (Figure 2). Chi-square tests of independence indicated statistically significant differences between country and riding school's offered education in BW [χ^2^ (1, *N* = 568) = 11.2, *p* = <.001] and LC [χ^2^ (1, *N* = 568) = 22.3, *p* = <.001], indicating that Swedish riding schools were more likely to offer education in both subjects.

**Figure 3:**
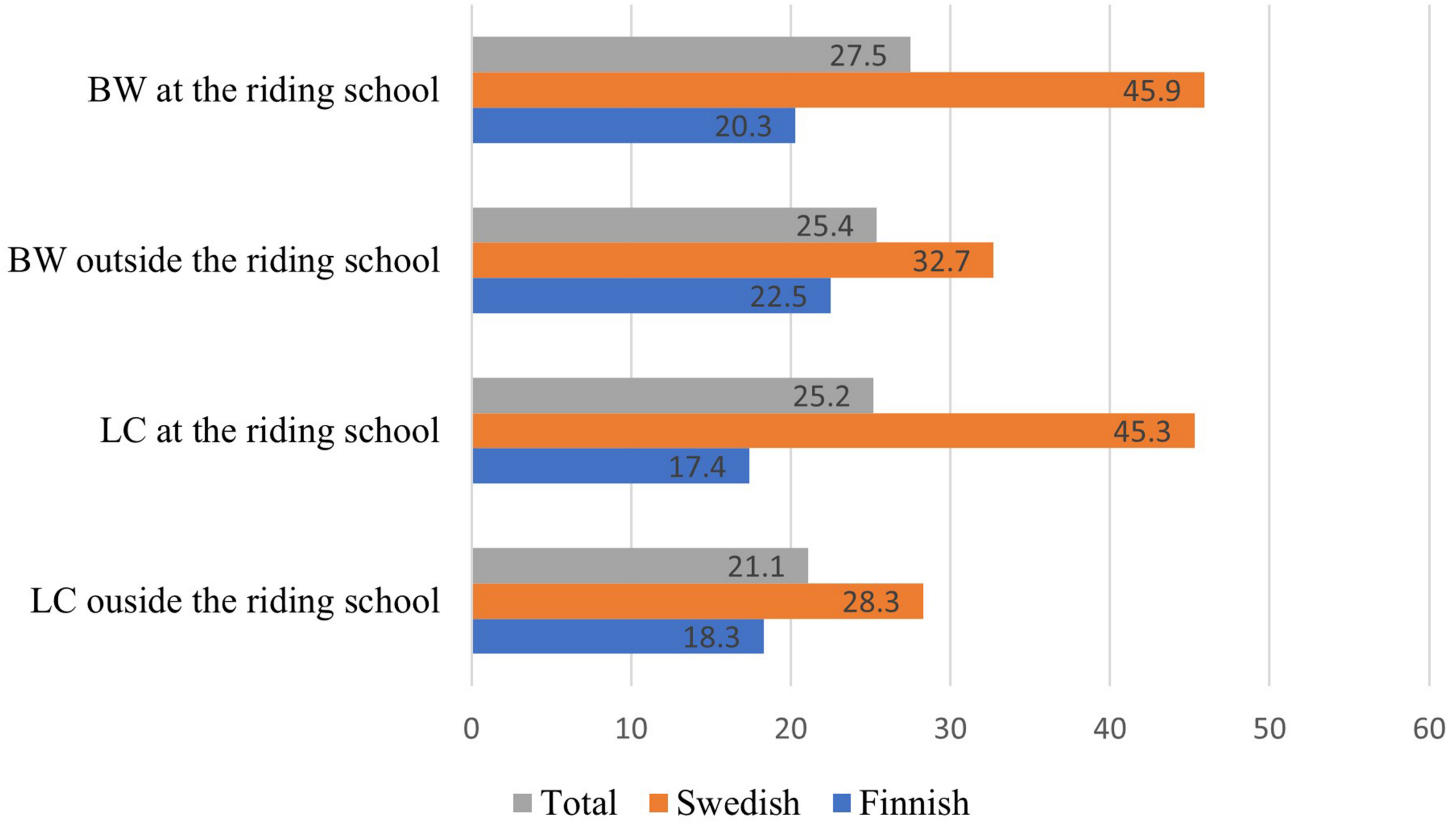
Illustration of participation in education in BW and LC during the last two semesters. Numbers are presented in percent (%).

Of all respondents, 27% had participated in education in BW and 25% in LC during the last two semesters at the riding school (Figure 3). There was a significant difference between Finnish and Swedish respondents' participation in education in BW [χ^2^ (1, *N* = 568) = 37.7, *p* = <.001], as well as in LC [χ^2^ (1, *N* = 568) = 47.4, *p* = <.001] at the riding school. A significant difference between the countries regarding participation in BW and LC outside the riding school (χ^2^ (1, *N* = 568) = 6.3, *p* = .012, χ^2^ (1, *N* = 568) = 6.8, *p* = .009, respectively) was also found. The results indicate that Swedish respondents had participated in education in both subjects to a greater extent than the Finnish respondents.

### 3.3. Riding school pupils’ motivational and amotivational regulation

The means, medians, and standard deviations for all motivational and amotivational regulations are listed in Table 2.

**Table 2:** Means, medians, standard deviations, range, and Cronbach's alpha for the sum variables.

	M	Mdn	SD	Min	Max	*α*
Intrinsic/Identified regulation	4.35	4.50	.70	1	5	.88
Integrated regulation	3.64	3.67	1.06	1	5	.84
Controlled motivation	1.48	1.40	.54	1	4	.64
Autonomous motivation^a^	4.12	4.22	.75	1	5	.90
Amotivation value/task	1.51	1.25	.63	1	4	.87
Amotivation ability	1.21	1.00	.46	1	5	.77
Amotivation effort	2.10	2.00	1.00	1	5	.73
Total amotivation^b^	1.61	1.50	.56	1	4	.87
Autonomy satisfaction	3.84	4.00	.70	1	5	.80
Competence satisfaction	4.04	4.14	.64	2	5	.76
Relatedness satisfaction	4.08	4.25	.72	1	5	.87
Total needs satisfaction^c^	3.99	4.09	.61	2	5	.92
Perceived horse welfare	4.13	4.50	.99	1	5	.84

^a^ Average of intrinsic/identified and integrated regulation.

^b^ Average of value/task, ability, and effort variables.

^c^ Average of autonomy, competence, and relatedness variables.

Intrinsic/identified regulation of motivation received the highest mean score (4.35) and controlled motivation the lowest (1.48) on a Likert scale from 1 to 5. This indicates that respondents participate in education in BW and LC at the riding school because it is interesting and personally important. Regarding amotivation, effort received the highest score (2.10) and ability the lowest (1.21), indicating that amotivation was uncommon, but experiencing amotivation due to lack of time and/or energy was the most prominent factor of it.

The variables autonomous motivation (an average of intrinsic/identified and integrated regulation), total amotivation (an average of value/task, effort, and ability), and controlled motivation were used to examine differences between Finnish and Swedish respondents. A Mann-Whitney test revealed that Swedish respondents had a significantly higher score on autonomous motivation (*U* = 24,051.5, *z* = −4.84, *p* < .001), a lower score on controlled motivation (*U* = 26,969.0, *z* = −3.24, *p*.001), and a lower score on total amotivation (*U* = 20,633.5, z = −6.79, *p* < .001) compared with Finnish respondents. This indicates that Swedish respondents felt more autonomously motivated and experienced less amotivation to participate in education in BW and LC than Finnish respondents.

### 3.4. Basic psychological needs satisfaction at the riding school

Among the respondents, relatedness received the highest score (4.08) and autonomy the lowest (3.85) on a Likert scale from 1 to 5. These results indicate that the respondents experienced a sense of belonging and accomplishment at the riding school, and to a lesser degree, a sense of volition.

A multiple linear regression analysis was used to estimate the relationship between autonomous motivation to participate in BW and LC and psychological needs satisfaction at the riding school, while controlling for age, hobby years (how long they had participated in the hobby), and hobby frequency (how often they participate in the hobby). To conduct the analysis, the variables total needs satisfaction (total score based on an average of autonomy, competence, and relatedness) and autonomous motivation were used. The overall regression was statistically significant [R^2^adj = .05, *F*(4, 563) = 8.45, *p* = <.001], and basic needs satisfaction significantly predicted autonomous motivation score (*β* = .224, *p* = <.001). Hobby years was found to negatively predict autonomous motivation score (*β* = −.088, *p* = .040), whereas hobby frequency and age did not predict autonomous motivation score.

### 3.5. The effect of background factors on inspected variables

Differences between groups regarding autonomous motivation, controlled motivation, total amotivation, and autonomy, competence, and relatedness needs satisfaction were also examined. The inspected groups were based on respondents' age, hobby years (how long they had participated in the hobby), hobby frequency (how often they participate in the hobby), and competition (if they had competed during the last two years or not.

Mann-Whitney tests indicated that competing respondents had a significantly higher score on controlled motivation (*U* = 32,738.5, *z* = −2.64, *p* = .008) and competence satisfaction (*U* = 33,308.0, *z* = −2.27, *p* = .023), and a lower score on autonomous motivation (*U* = 33,619.0, *z* = −2.11, *p* = .035) than non-competing respondents. This result suggests that competing respondents experienced less autonomous motivation and more controlled motivation towards participating in education in BW and LC, and more competence satisfaction at the riding school. Further, respondents that had participated in the hobby more often (hobby frequency) had a significantly higher score on competence (*U* = 33,272.5, *z* = −3.28, *p* = .001) and on relatedness (*U* = 34,713.0, *z* = −2.53, *p* = .011) compared with respondents that had participated less often. There were no significant differences regarding hobby frequency and motivational regulation, amotivation, or autonomy satisfaction. Also, there were no differences between competing and non-competing respondents regarding amotivation, autonomy, or relatedness satisfaction.

A Spearman correlation coefficient showed that there was a significant negative correlation between age group and controlled motivation [*r*(566) = −.22, *p* = <.001] and age group and amotivation [*r*(566) = −.16, *p* = <.001], suggesting that younger respondents experienced more controlled motivation and more amotivation towards participating in education in BW and LC than older respondents. There was also a significant negative correlation between hobby years and controlled motivation [*r*(566) = −.15, *p* = <.001], and a positive correlation between hobby years and competence [*r*(566) = .14, *p* = <.001], indicating that respondents that had practiced the hobby for a shorter time experienced more controlled motivation towards participating in education in BW and LC, and less competence satisfaction at the riding school. No statistically significant correlations were found between age group and autonomous motivation or any of the three needs. Further, no significant correlations were found between hobby years and autonomous motivation, amotivation, or autonomy and relatedness satisfaction.

### 3.6. Additional questions

Respondents' perceived inability to pay for education in BW and LC and experience of horse welfare at the riding school were also examined. Of all respondents (*N* = 568), the mean score for inability to pay was 1.96 (SD = 1.10) on a Likert-type scale from 1 to 5, suggesting that money was not a critical reason for non-participation. The mean score for perceived horse welfare at the riding school was 4.13 (SD = .99), indicating that the respondents experienced horses to be cared for and handled well at the riding school. Mann-Whitney tests suggested that the Finnish respondents were more likely than the Swedish to experience inability to pay for education (*U* = 20,171.0, *z* = −7.59, *p* < .001). Further, Spearman's correlation showed a significant negative correlation between age group and inability to pay [*r*(566) = .15, *p* = <.001] and a significant positive correlation between age group and perceived horse welfare [*r*(566) = .13, *p* = .003]. This shows that younger respondents were more likely to experience inability to pay for education in BW and LC, and less likely to experience that horses were cared for and handled well at the riding school. There was also a significant negative correlation between hobby years and inability to pay [*r*(566) = .14, *p* = <.001], suggesting that respondents who had practiced the hobby for a shorter time experienced more inability to pay for education. There were no significant differences between country and perceived horse welfare, or between competing and non-competing respondents regarding inability to pay or perceived horse welfare. Further, there were no statistical correlations between hobby years and perceived horse welfare.

## 4. Discussion

The purpose of this study was to investigate Finnish and Swedish riding school pupils' participation in non-riding education in horse behavior and welfare (BW) and learning and human-horse communication (LC) at the riding school. The results showed that forty percent of the Finnish and Swedish riding schools offered education in BW, and thirty-two percent in LC. Of all respondents, twenty-seven percent had participated in education in BW at the riding school, and twenty-five percent in LC. Furthermore, the results indicate that the respondents were autonomously motivated to participate in BW and LC, i.e., they participate because it is interesting and personally important. In addition, the riding school pupils' perceived satisfaction of the basic psychological needs for autonomy, competence, and relatedness at the riding school were explored. It was found that the need for relatedness was most satisfied, followed by competence, and that needs satisfaction predicted autonomous motivation to participate in education in BW and LC. Throughout the results of this study, Finnish riding schools offered less education, and Finnish respondents reported less participation in education, less autonomous motivation to participate, and lower relatedness and competence satisfaction.

The finding that less than half of the riding schools offered non-riding education in BW and LC, and that even fewer respondents had participated in education, is alarming, as knowledge and skills in BW and LC have been identified as very important both for horse welfare and human safety (14–16). Most riders receive their basic education in riding and horsemanship at the riding school, therefore, the importance of this educational platform cannot be ignored. In this study, Swedish riding schools were more likely to offer, and Swedish respondents were more likely to have participated in education in BW and LC. An explanation for the latter finding may be that Swedish riding schools include at least some compulsory non-riding education for all riders (38), whereas Finnish riding schools do not. As this study investigated only non-riding education, it is possible that education in BW and LC are included in the riding lessons at the riding schools. However, previous studies indicate that education in riding is focused on the technical aspects of riding, e.g., teaching pupils how to sit on the horse and how to control it (21, 25).

In this study, the Finnish and Swedish riding school pupils were autonomously motivated to participate in non-riding education in BW and LC at the riding school. The main motivational regulation was intrinsic/identified, meaning that they participated, or would participate, because it is fun, interesting, personally valuable, and a means to learn important things. Previous research from the equestrian domain suggest that riders are autonomously motivated, but these studies investigated riding activity, not non-riding education (58, 59, 61). In this study, integrated regulation, i.e., participating because it is a part of one's identity, stood out as a sole construct. This is interesting from a theoretical perspective, as it contrasts with recent meta-analyses testing the constructs of motivation in several domains (50, 74). Perhaps integration, a process where values and behaviors are merged into the self (42), is a distinct construct only in some specific contexts. Indeed, previous research on the riding school culture in Finland and Sweden have emphasized the strong identity formation that occurs among adolescent riders (27, 62, 75). Unfortunately, this study's scope did not include an investigation of the effects of the motivational constructs.

Findings in this study point towards groups that may be less autonomously motivated. Especially younger respondents reported more controlled motivation, i.e., internal or external pressure to participate, and more amotivation, i.e., lack of motivation. Also, competing respondents and respondents that had practiced the hobby for a shorter period reported more controlled motivation. Possible reasons for these results may be numerous, however, one potential reason is that, for these groups, the internalization process has not yet developed fully and the groups have not yet taken in the importance and value of participating in education in BW and LC. Internalization is a sort of a socialization process in which extrinsic motives, such as participation in education that is not perceived as innately enjoyable, are incorporated with personal meaning or integrated into the self and thus enables autonomous motivation (42, 48). However, competing respondents are likely to have actively practiced the hobby for a longer time and supposedly should have internalized values and practices. This raises the question of whether non-riding education in BW and LC at the riding school is considered important and valuable within this group and whether the education available meets the needs of this group. To facilitate internalization, practitioners such as riding teachers can provide rational that is relevant to the riders and autonomy support, i.e., take the perspective of the learner, be open and accepting (48, 76).

Further, satisfaction of the basic psychological needs is essential for the internalization process and autonomous motivation (42, 48). Feeling volitional, capable, and connected to others in one context, e.g., at school, may also influence motivation at the situational level, such as during class (77, 78). The finding that perceived needs satisfaction at the riding school in general predicted autonomous motivation to participate in non-riding education in BW and LC supports these assumptions and highlights the importance of needs satisfaction at the riding school. Besides fostering internalization of motives to participate in non-riding education, needs satisfaction is crucial for riders to continue with the hobby at the riding school. Previous research indicates that needs frustration, specifically autonomy, at the riding school is a major motive for riders to buy a horse of their own, i.e., quit at the riding school (60). As autonomy satisfaction received the lowest score in the present study, this raises the prospect of riding schools to become more autonomy supportive, to ensure internalization of important behaviors and lasting client relationships.

Finnish and Swedish riding school pupils' responses were found to differ significantly in almost all aspects of this study. Finnish respondents reported less participation in education, higher levels of controlled motivation and amotivation, and lower levels of autonomous motivation as well as competence and relatedness satisfaction. Further, Finnish riding schools were reported to offer less education in both BW and LC. The riding schools in both countries are very similar due to a comparable background and structure (28, 29, 31, 33, 79), however, some small differences also exist. For example, Swedish riding schools, in contrast to Finnish, are mostly run by non-profit clubs, have larger riding groups, and include some compulsory non-riding education for all riders (30, 38). These differences may influence the price of the activities, and thus explain the Finnish respondents’ higher reported inability to pay for extra education. Moreover, as discussed before, needs satisfaction, autonomous motivation, and engagement work conjointly within the process of internalization (42, 48, 52). Hence, merely offering non-riding education in BW and LC, for example as an integral part of the hobby for all riders, may be sufficient to highlight its value and importance and thus increase autonomous motivation to participate. As the national equestrian federations have some impact on the organization of non-riding education at riding schools (31, 80), they could highlight the importance of BW and LC as essential educational subjects at riding schools and support riding schools in providing such education.

### 4.1. Limitations and suggestions for future research

This study was based on a convenience sample; therefore, the main limitation is generalizability and sample bias. Perhaps riding school pupils with an interest in the subject were more likely to take part compared to non-interested pupils. However, due to an overall large sample (a total of 568 respondents), the findings are likely to represent many riding school pupils, and the internal relations are generalizable within the sample. The sample was gendered (96,5%), but so is the equestrian sport in Finland (94%–97% female) and Sweden (92% female) (26, 79).

Another limitation was the scales used for measuring motivation, amotivation, and needs satisfaction. They were not previously designed or validated in this specific equestrian context, and not back-translated for use in the current study. Furthermore, the BPNS-W scale (67–69) has received some criticism particularly due to low construct invariance (81, 82). Nevertheless, the scale was chosen because it was considered as more appropriate for the context than other available scales and needed less adjustments to the items. To mitigate the afore mentioned limitations, all items were tested with a group of riding school pupils, and the translations were checked by a researcher familiar with the theory and another person fluent in all three languages. The concepts used in the questionnaire were loosely operationalized, therefore, the respondents were likely to have various understanding of specifically education, horse behavior, welfare, learning, and human-horse communication. This may have influenced both validity and reliability of the study. Finally, it is acknowledged that education in these subjects may already be included in the riding lessons, however, this was not within the scope of this paper to investigate.

As this study was the first, to our knowledge, to investigate riders' motivation towards participation in non-riding education, further research is required to confirm these results. Specifically, as this study may have been subject to sample bias, future research could use probability sampling methods. To allow for instructional and situational factors to be investigated, future research could consider measuring motivation in connection with an intervention study. The present study focused on riding school pupils’ motivation and education at the riding school, however, the knowledge gap extends beyond this context. Mapping education available today outside of the riding school, together with analyses of participants' demographic backgrounds, motives, and educational contents is needed to adequately address the issue of equestrians' insufficient knowledge about the horse, and to ensure both horse welfare and human safety.

## Data Availability

The raw data supporting the conclusions of this article will be made available by the authors, without undue reservation.
