# Supplementary material for: Finnish and Swedish riding school pupils' motivation towards participation in non-riding education
Source: Front Sports Act Living. 2023 Oct 20;5:1232428. doi: 10.3389/fspor.2023.1232428 (PMC10622966; doi:10.3389/fspor.2023.1232428)
Supplement: Supplementary file 1 [file Table1.docx]

Supplementary material S1. Factor loadings and communalities for the motivational and amotivational variables, extracted with principal axis factoring, rotation method oblimin with Kaiser normalization.

|  |  | |  |  |  |  |  |
| --- | --- | --- | --- | --- | --- | --- | --- |
|  | 1 | 2 | 3 | 4 | 5 | 6 | Communality |
| I participate because it is interesting | -0.69 |  |  |  |  |  | 0.69 |
| I participate because it is a good way to learn things which could be useful to me | -0.67 |  |  |  |  |  | 0.69 |
| I participate because it’s important to me | -0.64 |  |  |  |  |  | 0.68 |
| I participate because it’s fun | -0.59 |  |  |  |  |  | 0.63 |
| I participate because I find it pleasurable | -0.58 |  |  |  |  |  | 0.41 |
| I participate because I value the benefits of the education | -0.43 |  |  |  |  |  | 0.49 |
| I participate because if I don’t other people will not be pleased with me |  | 0.55 |  |  |  |  | 0.37 |
| I participate because I would feel guilty if I didn’t |  | 0.55 |  |  |  |  | 0.40 |
| I participate because other people say that I should |  | 0.55 |  |  |  |  | 0.37 |
| I participate because I feel obligated to participate |  | 0.54 |  |  |  |  | 0.30 |
| I participate because people around me reward me when I do |  | 0.37 |  |  |  |  | 0.17 |
| I participate because I feel better about myself when I do |  |  |  |  |  |  | 0.41 |
| I do NOT participate because I would learn barely anything new |  |  | 0.66 |  |  |  | 0.50 |
| I do NOT participate because, for me, it holds no interest |  |  | 0.63 |  |  |  | 0.70 |
| I do NOT participate because I have the impression that it’s always the same thing every time |  |  | 0.60 |  |  |  | 0.55 |
| I do NOT participate because I don’t want to spend my time on it |  |  | 0.56 |  |  |  | 0.62 |
| I do NOT participate because it is not important to me |  |  | 0.53 |  |  |  | 0.53 |
| I do NOT participate because I find that participating is boring |  |  | 0.53 |  |  |  | 0.53 |
| I have NO good reason to participate |  |  | 0.51 |  |  |  | 0.41 |
| I do NOT want to pay for education |  |  | 0.48 |  |  |  | 0.38 |
| I do NOT have the time to participate |  |  |  | 0.68 |  |  | 0.61 |
| I do NOT participate because I’m not energetic enough |  |  |  | 0.39 |  |  | 0.48 |
| I CANNOT afford to participate |  |  |  |  |  |  | 0.18 |
| I do NOT have the knowledge/skills required to participate |  |  |  |  | 0.69 |  | 0.52 |
| I do NOT participate because the tasks demanded surpass my abilities |  |  |  |  | 0.67 |  | 0.55 |
| I do NOT participate because I’m not good at it |  |  |  |  | 0.60 |  | 0.50 |
| I participate because it reflects who I am |  |  |  |  |  | -0.93 | 0.80 |
| I participate because it’s a part of who I am |  |  |  |  |  | -0.64 | 0.64 |
| I participate because it allows me to live in line with my values |  |  |  |  |  | -0.56 | 0.58 |
